# In this issue: Volume 114, Issue 7, July 2023

**DOI:** 10.1111/cas.15885

**Published:** 2023-07-05

## 1 The histone demethylase Utx controls CD8+ T‐cell‐dependent antitumor immunity via epigenetic regulation of the effector function


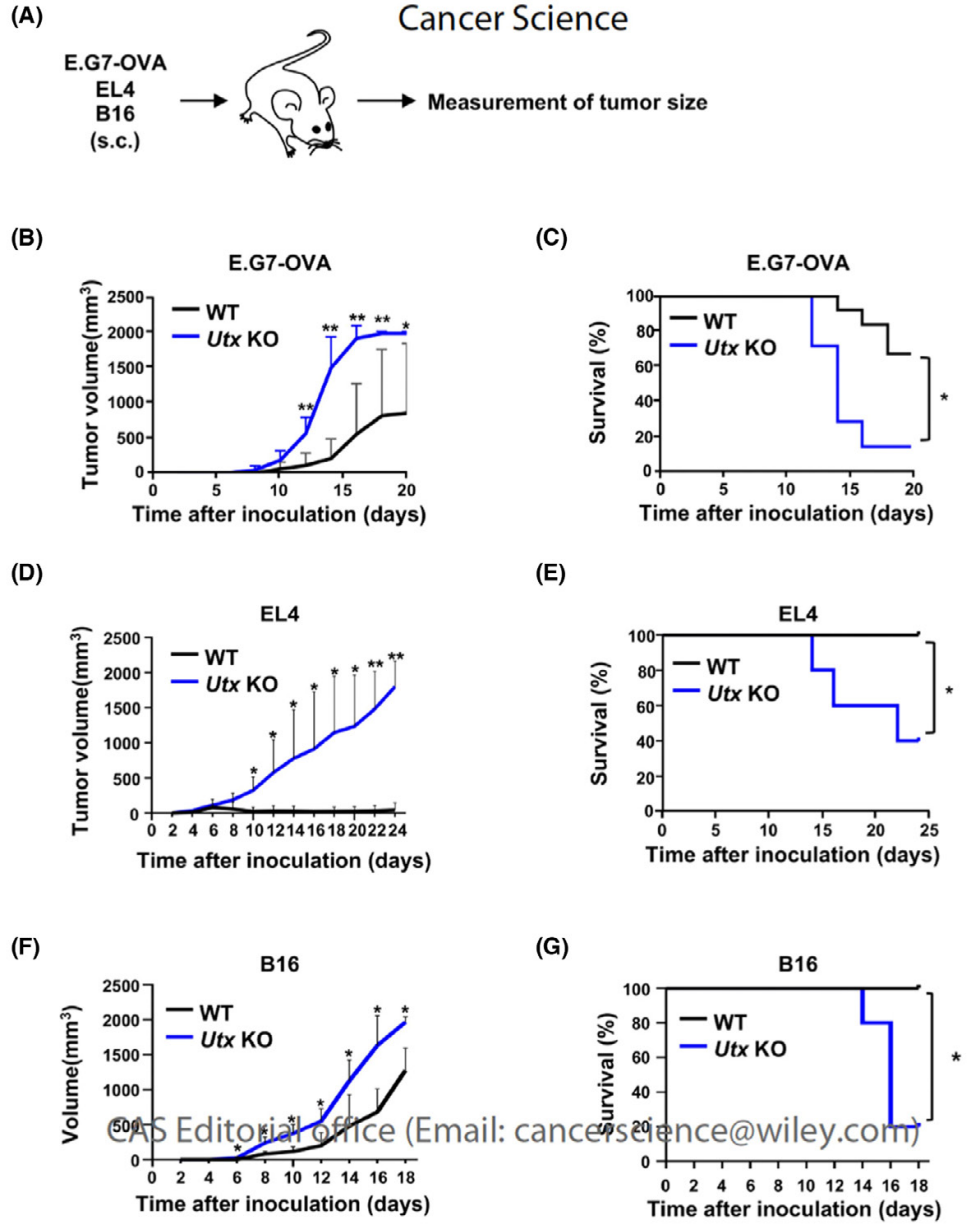
Cytotoxic T‐lymphocytes (CD8+ T cells) play an important role in the body's immune defense against tumors. When inactive CD8+ T cells identify specific antigens, they activate and transform into functional CD8+ T cells. These functional cells migrate to the tumor site and play a vital role in destroying tumors and supporting the body's anti‐tumor immune response.

However, specific genes need to be activated or deactivated in order to ensure that these cells function properly. This control over gene expression is achieved through the process of epigenetic regulation which involves modifications to DNA and histones (proteins that help package DNA) in CD8+ T cells through the addition of chemical molecules such as methyl groups, i.e., methylation.

In the current study, researchers investigated the role of Utx, an enzyme that removes chemical modifications like methylation from the histone – H3K27. The team sought to study its influence on the development of CD8+ T cells and their ability to fight tumors. This was achieved by comparing tumors in mice that had T cells lacking Utx with the tumors in normal mice.

Results from the study showed that the mice that lacked Utx exhibited larger tumors and lower survival rates when compared with normal mice. They also observed that the number of CD8+ T cells within the tumor‐infiltrating lymphocytes (TILs) was significantly reduced in mice lacking Utx. Further analysis revealed that CD8+ T cells lacking Utx took longer to develop into their active form and proved less effective against tumors.

Upon assessing the genes of these cells, it was found that the absence of Utx in these cells led to a disruption in the normal gene expression patterns associated with CD8+ T cell activation and function. Furthermore, the study revealed that CD8+ T cells lacking Utx showed reduced expression of the CXCR3 (chemokine receptor) gene, which plays a crucial role in their migration to tumor sites.

Overall, these findings indicate that Utx helps strengthen immune response against tumors through the process of epigenetic regulation and together with cellular metabolism, regulate the function of activated CD8+ T cells.

Understanding the epigenetic regulation of CD8+ T cell responses and chemokine receptor expression may lead to the development of more effective cancer treatments. Thus, manipulating the gene expression profiles of T cells through epigenetic interventions shows promise in improving the therapeutic outcomes and prognosis of patients with cancer.

https://onlinelibrary.wiley.com/doi/full/10.1111/cas.15814

## 2 A gain‐of‐function mutation in microRNA 142 is sufficient to cause the development of T‐cell leukemia in mice


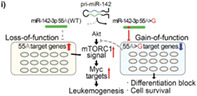


MicroRNAs (miRNAs) are small genetic (RNA) molecules that play an important role in regulating gene expression. In cancer cells, these molecules commonly undergo alterations like mutations and chemical modifications. However, until now, there have been no reports to suggest that the mutations in miRNAs result in cancer development.

Research reveals that a specific miRNA called miR‐142 is often mutated in cancers like lymphoma and leukemia. In a recent study, Kawano et al sought to investigate the role of miR‐142 mutations in the development of leukemia. To achieve this, they used a powerful gene‐editing tool called CRISPR‐Cas9. They employed this tool on mice to introduce a particular mutation in miR‐142, which is frequently seen in patients with acute myeloid leukemia/myelodysplastic syndrome (AML/MDS).

The researchers subsequently harvested bone marrow cells from these mutant mice and transplanted them into normal mice. They found that mice with the miR‐142 mutation developed CD8+ T cell leukemia after the transplantation.

In order to understand how miR‐142 causes leukemia, the authors performed a genetic analysis of stem cells and CD8+ T cells from the mutant mice. This analysis was carried out before and after leukemia onset to identify the molecular changes that give rise to cancer.

Findings from the study revealed that certain genes, which were more active in the leukemia‐causing CD8+ T cells, belonged to the mTORC1 and MYC pathways that regulate cell growth, division, and metabolic processes, and were found to drive cancer growth. Additionally, it was revealed that the genes involved in cell death, cell differentiation, and the inhibition of the Akt–mTOR pathway were less active in the CD8+ T cells of the mutant mice.

In summary, the miR‐142 mutation both reduced the function of normal miR‐142 and gained the ability to suppress specific genes, leading to the development of leukemia in mice. In conclusion, this study offers valuable insights into the role of miRNAs in the development of blood cancer.

https://onlinelibrary.wiley.com/doi/full/10.1111/cas.15794

## 3 RHAMM marks proliferative subpopulation of human colorectal cancer stem cells


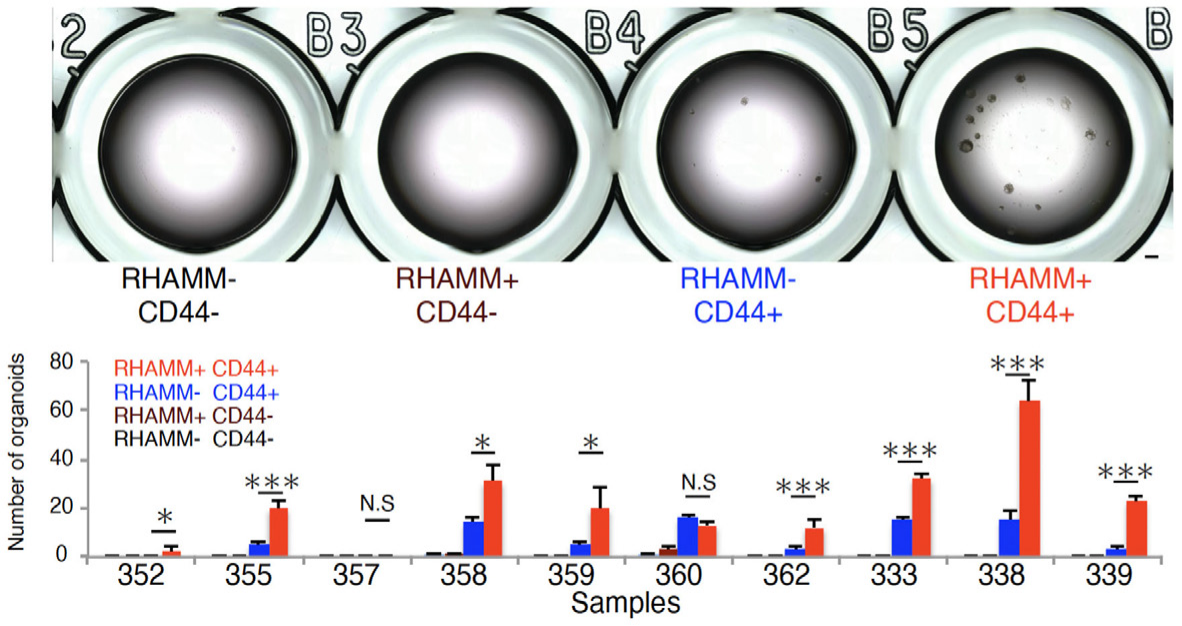


Cancer stem cells (CSCs) are a special subgroup of cells within tumors that have the ability to self‐renew and differentiate into various cell types found in the tumor. They are believed to play a crucial role in tumor growth, progression, and resistance to treatment. To better understand and develop new treatments for tumors, it is important to identify molecules that are characteristic of CSCs. These characteristics or substances (like proteins, genes, hormones, enzymes, or other molecules) are found on or within cancer cells and can be detected and measured. However, isolating cancer stem cells from the human body is currently difficult due to the lack of a defining molecular marker for their identification.

In this study, researchers aimed to find specific markers that can identify CSCs within heterogeneous tumors, using organoids which are small versions of tumor tissues grown in the lab. They identified a specific subgroup of CD44+ cells in colorectal cancer stem cells, characterized by the presence of a cell surface protein, RHAMM (receptor for hyaluronan‐mediated motility). These cells had distinct characteristics compared to other cell types. To confirm their findings, the team isolated RHAMM‐positive cells from actual human colorectal cancer tissues. These cells showed high proliferation and the ability to renew themselves. The study also found that inhibiting RHAMM strongly prevented the formation of organoids in the lab and inhibited tumor growth in animal models.

These study findings suggest that RHAMM is required for maintaining the proliferative characteristics of CSCs and the presence of RHAMM is a unique characteristic of the actively dividing subgroup of CSCs within CD44+ colorectal cancer. Therefore, RHAMM could be a potential therapeutic target for developing new therapies for human colorectal cancer. However, targeting only the RHAMM+ subpopulation may have limitations owing to tumor heterogeneity; hence, a multitarget strategy may be required to eradicate all cancer cells.

https://onlinelibrary.wiley.com/doi/full/10.1111/cas.15795

